# Levels of acute phase proteins remain stable after ischemic stroke

**DOI:** 10.1186/1471-2377-6-37

**Published:** 2006-10-16

**Authors:** Mitchell SV Elkind, Kristen Coates, Wanling Tai, Myunghee C Paik, Bernadette Boden-Albala, Ralph L Sacco

**Affiliations:** 1 Gertrude H. Sergievsky Center, College of Physicians and Surgeons, Columbia University, New York, NY, USA; 2 Department of Neurology, College of Physicians and Surgeons, Columbia University, New York, NY, USA; 3 Department of Biostatistics, Mailman School of Public Health, Columbia University, New York, NY, USA; 4 Department of Sociomedical Sciences, Mailman School of Public Health, Columbia University, New York, NY, USA; 5 Division of Epidemiology, Joseph P. Mailman School of Public Health, Columbia University, New York, NY, USA

## Abstract

**Background:**

Inflammation and inflammatory biomarkers play an important role in atherosclerosis and cardiovascular disease. Little information is available, however, on time course of serum markers of inflammation after stroke.

**Methods:**

First ischemic stroke patients ≥40 years old had levels of high-sensitivity C-reactive protein (hsCRP), serum amyloid A (SAA), and fibrinogen measured in plasma samples drawn at 1, 2, 3, 7, 14, 21 and 28 days after stroke. Levels were log-transformed as needed, and parametric and non-parametric statistical tests were used to test for evidence of a trend in levels over time. Levels of hsCRP and SAA were also compared with levels in a comparable population of stroke-free participants.

**Results:**

Mean age of participants with repeated measures (n = 21) was 65.6 ± 11.6 years, and 13 (61.9%) were men, and 15 (71.4%) were Hispanic. Approximately 75% of patients (n = 15) had mild strokes (NIH Stroke Scale score 0–5). There was no evidence of a time trend in levels of hsCRP, SAA, or fibrinogen for any of the markers during the 28 days of follow-up. Mean log(hsCRP) was 1.67 ± 1.07 mg/L (median hsCRP 6.48 mg/L) among stroke participants and 1.00 ± 1.18 mg/L (median 2.82 mg/L) in a group of 1176 randomly selected stroke-free participants from the same community (p = 0.0252).

**Conclusion:**

Levels of hsCRP are higher in stroke patients than in stroke-free subjects. Levels of inflammatory biomarkers associated with atherosclerosis, including hsCRP, appear to be stable for at least 28 days after first ischemic stroke.

## Background

Basic and clinical studies provide evidence that inflammation plays a crucial role in atherosclerosis and cardiovascular disease [1]. Despite a growing literature on the role of acute phase proteins, particularly high-sensitivity C-reactive protein (hsCRP), and other inflammatory markers in risk stratification and prediction of outcomes among patients with cardiovascular disease [2-6], very little is known about the role of these markers in predicting outcome in patients with cerebrovascular disease. Some investigators have suggested that levels of acute phase reactants predict prognosis after stroke [7-13], although a recent review concluded that there was insufficient data available to recommend testing for these markers in stroke patients at present [14]. Little information is available, however, on the time course of serum markers of inflammation and related proteins after stroke. This consensus statement [14] also concluded that further studies were needed to determine the time course of levels of these markers after stroke.

Our objective was to determine the stability of levels of inflammatory biomarkers after ischemic stroke. We measured plasma levels of acute phase proteins at seven pre-specified time points up to 28 days in patients with ischemic stroke and assessed for trends in these values. We also compared these levels to those in a comparable stroke-free population.

## Methods

The Northern Manhattan Study (NOMAS) includes a population-based incident ischemic stroke follow-up study in a multi-ethnic, urban population. The methods of patient identification and enrollment have been described in previous publications [15-17]. Briefly, stroke patients were enrolled if they: (1) were diagnosed with a first stroke; (2) were over age 40, and (3) resided in Northern Manhattan for ≥3 months in a household with a telephone. For this analysis, a subsample (n = 21) of hospitalized ischemic stroke patients who had blood collected at seven pre-specified time intervals after stroke were included [17]. The study was approved by the CUMC Institutional Review Board. All participants gave consent directly or through a surrogate when appropriate.

Data were collected through interviews by trained research assistants, and physical and neurological examinations were conducted by study neurologists, as previously described [15-17]. When possible, data were obtained directly from subjects using standardized data collection instruments. When the subject was unable to provide answers, a proxy knowledgeable about the subject's history was interviewed. Race-ethnicity was based upon self-identification. Medical history was determined using standardized questions adapted from the Behavioral Risk Factor Surveillance System from the Centers for Disease Control and Prevention [18]. Hypertension was defined as a history of hypertension or use of anti-hypertensive medications, and diabetes mellitus was defined by a fasting blood glucose level ≥126 mg/dl, the subject's self-report of such a history, or insulin or oral hypoglycemic use.

Stroke diagnostic evaluation included computerized tomography and/or magnetic resonance imaging of the brain, ultrasound evaluation of the extracranial and intracranial cerebral vessels, and transthoracic or transesophageal echocardiogram as appropriate. Assessment of stroke subtype using modified TOAST (Trial of Org 10172 in Acute Stroke Treatment) criteria was determined by a consensus of stroke neurologists, using all available information, as described in a previous publication [19]. Baseline stroke severity was assessed using a derived National Institutes of Health Stroke Scale (NIHSS), and categorized as mild (0–5), moderate (6–13) or severe (≥14) [16].

### Measurement of acute phase proteins

Both serum and plasma (in EDTA) samples were collected in the morning at days 1, 2, 3, 7, 14, 21, and 28 after stroke onset. The mean number of hospital days for the patient sample was 11.7 ± 18.7 days and 74 samples were drawn in the hospital and the remainder was collected after discharge. All blood tubes were centrifuged at 3000 g for 10 minutes. Serum and plasma were immediately separated, aliquoted and stored at -70°C until specimens could be assayed. High sensitivity C-reactive protein (hsCRP), serum amyloid A (SAA) and fibrinogen were measured using plasma samples in the Columbia University Medical Center laboratory using the BNII system (Dade-Behring, Deerfield, IL). The intra-assay and inter-assay coefficients of variation were, respectively, as follows: for hsCRP, 1.8% and 4.5%; for SAA, 7.2% and 6.1%; and for fibrinogen, 1.6% and 5.7%.

### Statistical analyses

Statistical analyses were conducted using SAS Version 8.2 (SAS Institute, Cary, NC). Means and their standard deviations of hsCRP, SAA, and fibrinogen were calculated. Levels of all markers were log-transformed before analysis to stabilize the variance. Mean log-transformed values for each marker were compared across groups defined by demographic characteristics, stroke characteristics (NIHSS and diagnostic subtype), and risk factors. Tests for time trends were performed using both parametric and non-parametric methods. Random effects models were used to calculate the adjusted differences between mean values at each time point while taking the correlation among the repeated measurements into account. The best model for all outcomes was the model with linear, quadratic, and cubic time trends. Friedman's test was also used to test for statistical significance of differences between the markers at each time point without assuming multivariate normality. The Welch modified two-sample t-test was used to test for differences between the marker levels in the sample of stroke patients and a sample of stroke-free participants. Throughout, Type I error of the test was set as 0.05.

## Results

Table 1 lists the baseline demographic characteristics and vascular disease risk factor profile of the 21 participants. The mean age of the subjects was 65.6 ± 11.6 years, and 61.9% (n = 13) were male. The prevalence of vascular risk factors was high. Previous history of cardiovascular disease was also prevalent (38.1% had a history of coronary artery disease). Most had mild strokes (71.4%, n = 15), 23.8% (n = 5) had moderate strokes, and only 1 had a severe stroke.

There was no evidence of a time trend in levels of hsCRP, SAA, or fibrinogen for any of the markers during the 28 days of follow-up (Table 2, Figure). Results were similar using non-parametric tests. Although mean hsCRP increased from day 1 (8.08 ± 6.54 mg/L) to day 2 (16.42 ± 24.71 mg/L) and remained elevated throughout the period of follow-up (day 28: 20.89 ± 35.40 mg/L), this was not statistically significant.

In a random effect model adjusted for measurement day, age, sex, race-ethnicity, history of hypertension, diabetes mellitus, current smoking, coronary artery disease, stroke subtype, and stroke severity, log(hsCRP) was associated with current smoking (beta = 2.46 ± 1.22, p = 0.0469) and stroke severity (beta = 0.15 ± 0.08, p = 0.0539), but not with other risk factors. On day 1, mean log(hsCRP) was 1.54 ± 1.18 mg/L among current smokers versus 2.04 ± 0.58 mg/L among non-smokers. Mean log(hsCRP) was 1.54 ± 1.11 mg/L among those with mild stroke (NIH stroke scale <6) versus 2.04 ± 0.98 among those with moderate to severe stroke (NIH stroke scale ≥6).

In a random effect model SAA was associated with sex (p = 0.0017) and history of diabetes mellitus (p = 0.0349), but not with stroke severity, smoking, or other risk factors. Mean values of log(SAA) were 3.16 ± 0.55 mg/L for those with diabetes mellitus compared with 1.59 ± 1.14 mg/L for those without diabetes. Fibrinogen levels were not associated with patient characteristics.

In a group of 1176 stroke-free subjects (mean age 68.9 ± 10.0 years) drawn from the same underlying population as part of an ongoing prospective cohort study among stroke patients, mean levels of hsCRP were lower than the day 1 values in this population of stroke patients. The mean log(hsCRP) was 1.00 ± 1.18 (median hsCRP 2.82 mg/L) among stroke-free participants, and 1.67 ± 1.07 (median 6.48 mg/L) among stroke patients (p = 0.0252). Mean log(SAA) was not higher among stroke patients (1.78 ± 1.20 mg/L) than 1122 stroke-free participants (1.52 ± 0.80 mg/L; p = 0.4031). Median SAA among stroke patients was 5.70 versus 4.60 mg/L among stroke-free participants.

## Discussion

We found that levels of the acute phase proteins hsCRP, SAA, and fibrinogen are stable after stroke for at least one month in patients with their first mild to moderate ischemic strokes. There is no significant time trend in levels over this period of time. Although we did not have available levels of these proteins in these patients prior to their stroke, the levels of hsCRP in a comparable population of stroke-free participants was significantly lower, suggesting that hsCRP levels are higher in those with recent stroke. We think this is most likely because hsCRP levels increase at the time of stroke, although we cannot exclude the possibility that hsCRP is elevated in those stroke-free individuals who subsequently go on to develop stroke [2,14]. It thus appears that if levels of hsCRP increase at the time of stroke, they remain elevated for at least one month. Whether levels decrease after that time is not addressed by these data.

Several epidemiological studies provide evidence that inflammatory markers, particularly hsCRP, predict incident ischemic stroke in stroke-free populations [14]. Some investigators have provided evidence that inflammatory markers measured at the time of ischemic stroke predict future recurrent ischemic events [14], including stroke [13]. The prognostic significance of inflammatory markers after a vascular event has already occurred has been assessed in relatively few studies, however, particularly with regard to recurrent stroke as an outcome. Among stroke patients, IL-6 and IL-1 receptor antagonist, but not TNFα, measured at baseline were independent predictors of worsening in the first 24 hours after stroke [7,8]. Others found that hsCRP levels above 10.1 when measured within 72 hours of stroke predicted mortality over 4 years [10]. Others found that the measurement of CRP at 24 or 48 hours, but not at admission, also predicted outcome [12]. In one study, hsCRP levels ≥15 mg/L at discharge were associated with occurrence of a new vascular event or death at 1 year [11]. HsCRP levels in the highest quintile measured at least 3 months after a first ischemic stroke or TIA were associated with an increased risk of subsequent stroke or MI in another study [9]. In a nested case-control analysis of a large clinical trial population, elevated levels of both fibrinogen and hsCRP independently predicted recurrent ischemic stroke [13].

The time course of inflammatory markers and acute phase reactant proteins after ischemic events has been investigated in relatively few studies, particularly with regard to stroke. In one study [20], levels of fibrinogen and leukocyte count, but not hsCRP, were elevated up to one year after acute stroke compared to healthy control subjects. These data were limited to blood samples drawn at stroke onset, 6 weeks, 6 months, and 1 year after enrollment. In another study [21], levels of hsCRP measured at hospital discharge were more strongly associated with prognosis than measurements made at admission or between 48 and 72 hours. There was evidence of a change in hsCRP levels during hospitalization, but with some patients showing an increase and others a decrease in levels. Our data suggest that, on average, levels measured within the first month after stroke may not vary significantly, and that timing of measurement may not be crucial if levels within one month are used for prognostication. Currently, however, while several studies have indicated hsCRP may be used for purposes of prognostication [10-12], there is no consensus that this is appropriate [14].

We also found an association of post-stroke levels of hsCRP with smoking and stroke severity. Other studies have found an association between hsCRP levels and smoking in disease-free individuals [22-24], but few studies have evaluated this association in patients after stroke. In those studies in which this was assessed, no association was found [9-12]. We may have had greater power to detect associations, however, because of the greater number of measurements made at multiple time points. Many other inflammatory markers are similarly elevated in smokers [2], moreover, and this may provide a mechanism through which smoking increases the risk of vascular disease. Similar to some [10,11], but not all [9], other studies, we found an association of post-stroke hsCRP to stroke severity. Serum amyloid A has not been systematically studied in stroke patient populations.

Diabetes mellitus has also been associated with levels of hsCRP in cross-sectional analyses of stroke-free individuals [25,26], and the increased risk associated with elevated levels of hsCRP may be attenuated or absent in study participants with a history of diabetes [26,27]. Previous studies in stroke patients, however, have not demonstrated differences in hsCRP levels according to history of diabetes mellitus [9-12,21]. In studies that have found an effect of elevated hsCRP on risk of recurrent stroke, moreover, analyses were not adjusted for diabetes mellitus [13]. Further studies will be needed to determine whether the prognostic value of hsCRP after stroke is present in patients with diabetes mellitus.

Levels of inflammatory markers may remain elevated for at least a month after stroke for several reasons. First, patients with ischemic stroke may suffer ongoing complications, such as infections and deep venous thrombosis, which lead to persistent elevations in these markers. The patients in our study, however, were largely free of such complications and had milder strokes than would be expected among patients with these complications. Second, levels of acute phase reactants could be markers of an increased inflammatory state that first predisposed the patient to stroke, and we cannot exclude this possibility on the basis of our data. We found few correlations between levels of acute phase proteins and other vascular risk factors, however. Third, the cerebral or vascular injury caused by stroke and the subsequent recovery may lead to an upregulation in the production of these markers, and this process may persist for a month or longer [20]. Further studies in larger populations of patients, with correlation with stroke severity and markers of cerebral injury, are needed to resolve these questions.

A strength of our study is the availability of levels of several markers at multiple time points after ischemic stroke. Most studies have relied on a relatively small number of measurements. We also focused on patients with milder strokes, which limits the likelihood of confounding due to major medical complications.

There are also limitations to our study. Because of the requirement for frequent phlebotomy, we studied a relatively small number of participants, which limited our power to detect changes over time and between patient subgroups. We also do not have available measurements beyond 28 days. Future studies should assess these markers at longer time intervals after stroke.

## Conclusion

Levels of hsCRP are higher in stroke patients than in stroke-free subjects. Levels of inflammatory biomarkers associated with atherosclerosis, including hsCRP, appear to be stable for at least 28 days after first ischemic stroke. This information may be useful in planning future studies of the effect of inflammatory biomarkers on prognosis after stroke.

## Abbreviations

hsCRP = high-sensitivity C-reactive protein

MI = myocardial infarction

NIHSS = National Institutes of Health Stroke Scale

SAA = serum amyloid A

TIA = transient ischemic attack

TOAST = Trial of Org 10172 in Acute Stroke Treatment

## Competing interests

The author(s) declare that they have no competing interests.

## Authors' contributions

MSVE conceived of the study, participated in its design and coordination, and drafted the manuscript. KC supervised the immunoassays. WT performed the statistical analysis. MCP participated in the study design and coordination, and supervised the statistical analysis, providing important intellectual input. BBA participated in the study design and coordination. RLS conceived of the study, and supervised its design and coordination. All authors read and approved the final manuscript.

## Pre-publication history

The pre-publication history for this paper can be accessed here:



## Figures and Tables

**Figure 1:**
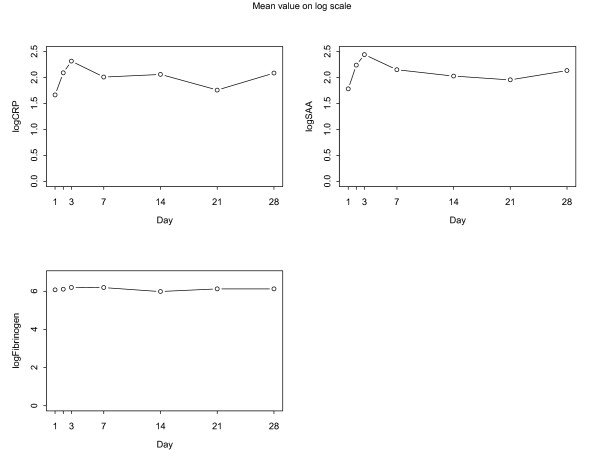
Mean values of log-transformed levels of acute phase proteins over time.

**Table 1:** Baseline characteristics of study population

Number of participants	21
Age, mean ± SD, y	65.6 ± 11.6
Male, N (%)	13 (61.9)
Race-ethnicity, N (%)	
Non-Hispanic White,	4 (19.1)
Non-Hispanic Black	2 (9.5)
Hispanic	15 (71.4)
Risk factors, N (%)	
History of coronary artery disease	8 (38.1)
Current smoking	4 (19.1)
Diabetes mellitus	3 (14.3)
Hypertension	14 (66.7)
Hypercholesterolemia	7 (33.3)
NIH Stroke Scale score, N (%)	
0–5	15 (71.4)
6–13	5 (23.8)
≥14	1 (4.8)
Stroke etiologic subtype, N (%)	
Atherosclerotic	2 (9.5)
Lacunar	7 (33.3)
Embolic	1 (4.8)
Cryptogenic	11 (52.4)

**Table 2:** Mean levels of log-transformed measures during 28 days of follow-up

**Day**	**N**	**log(CRP) mg/L**	**log (SAA) mg/L**	**log (fibrinogen) mg/dL**
**1**	16	0.72 ± 0.46	0.77 ± 0.51	2.64 ± 0.09
**2**	15	0.91 ± 0.53	0.97 ± 0.67	2.65 ± 0.11
**3**	20	0.99 ± 0.51	1.05 ± 0.71	2.70 ± 0.06
**7**	20	0.87 ± 0.56	0.93 ± 0.65	2.69 ± 0.08
**14**	19	0.90 ± 0.60	0.88 ± 0.60	2.60 ± 0.37
**21**	18	0.76 ± 0.61	0.85 ± 0.65	2.66 ± 0.12
**28**	17	0.91 ± 0.58	0.93 ± 0.64	2.66 ± 0.09
P*		0.7005	0.4764	0.6382

* p for trend over time using random effect models.
